# *De novo* leaf and root transcriptome analysis to identify putative genes involved in triterpenoid saponins biosynthesis in *Hedera helix* L.

**DOI:** 10.1371/journal.pone.0182243

**Published:** 2017-08-03

**Authors:** Huapeng Sun, Fang Li, Zijian Xu, Mengli Sun, Hanqing Cong, Fei Qiao, Xiaohong Zhong

**Affiliations:** 1 Key Laboratory of Crop Gene Resources and Germplasm Enhancement in Southern China, Ministry of Agriculture / Tropical Crops Genetic Resources Institute, Chinese Academy of Tropical Agricultural Sciences, Danzhou, China; 2 Horticulture & Landscape College, Hunan Agricultural University, Changsha, China; 3 Institute of Tropical Agriculture and Forestry, Hainan University, Haikou, China; Dokuz Eylul Universitesi, TURKEY

## Abstract

*Hedera helix* L. is an important traditional medicinal plant in Europe. The main active components are triterpenoid saponins, but none of the potential enzymes involved in triterpenoid saponins biosynthesis have been discovered and annotated. Here is reported the first study of global transcriptome analyses using the Illumina HiSeq^™^ 2500 platform for *H*. *helix*. In total, over 24 million clean reads were produced and 96,333 unigenes were assembled, with an average length of 1385 nt; more than 79,085 unigenes had at least one significant match to an existing gene model. Differentially Expressed Gene analysis identified 6,222 and 7,012 unigenes which were expressed either higher or lower in leaf samples when compared with roots. After functional annotation and classification, two pathways and 410 unigenes related to triterpenoid saponins biosynthesis were discovered. The accuracy of these *de novo* sequences was validated by RT-qPCR analysis and a RACE clone. These data will enrich our knowledge of triterpenoid saponin biosynthesis and provide a theoretical foundation for molecular research on *H*. *helix*.

## Introduction

*Hedera helix* L. is a perennially evergreen climbing vine that belongs to the Araliaceae family and is collected in the European Pharmacopoeia and British Pharmacopoeia as a traditional medicinal plant [1,2]. It occurs in Europe and is widely cultivated around the world. Functional components in *H*. *helix* with high medicinal value are extracted from fresh leaves and stems (European Pharmacopoeia 7.0, 2010). Clinical studies have revealed its pharmacological effect for treating cough [3], asthma [4], bronchitis [3,5,6] and other respiratory diseases. A recent pharmacology study also showed that the extracts have anti-inflammatory, antimicrobial, anti-oxidative, antitumour, anti-mutagenicity, antispasmodic, anti-leishmania and hepatoprotective activities [1]. Due to its efficacy, it had been approved by many European countries for clinical applications [7]. The extracts of *H*. *helix* can be made into several medicinal dosage forms, such as syrups and tablets. In Poland, preparations with its extracts as the main components are commercially available, including Hedelix syrups, Prospan, PiniHelix, Helical, Hederasal and Hederoin tablets [1,8].

Using modern biotechnology to dissect biosynthesis pathways and manipulate the functional genes involved in biosynthesis of the active components in medicinal plants are two main development directions for the modernization of herbal medicines. The main active components in *H*. *helix* are hederacoside C [9,10] and α-hederin [11,12], which belong to the oleanane-type triterpenoid saponins [13]. Many studies describe the biosynthesis pathway of triterpenoid saponins in three parts [14,15]. Part I is upstream isopentenyl diphosphate (IPP) and dimethylallyl pyrophosphate (DMAPP) biosynthesis [16,17], part II is the midstream carbocyclic of triterpenoid biosynthesis [18], and part III is the downstream modification pathway of complex functional groups in triterpenoid carbocyclic [19]. Genes upstream and midstream of the pathway have been intensively studied [20,21], but the specific downstream genes involved in catalysing triterpenoid saponin biosynthesis have yet to be revealed. Since genomic information for *H*. *helix* is lacking and no genes in the triterpenoid saponin pathway in this plant have been reported, transcriptome analysis using next-generation sequencing technology provides a convenient and economical tool. It is also powerful for identifying various transcripts and functional genes and for providing quantitative estimates of gene expression. Next-generation sequencing technology can also generate forty to eighty million bp of high-quality sequences per sample. It has been widely used in medicinal plants such as *Panax ginseng* [20], *Panax notoginseng* [21], *Asparagus racemosus* [22], *Cephalotaxus hainanensis* [23], *Pseudostellaria heterophylla* [24], *Sinopodophyllum hexandrum* [25], *Solanum elaeagnifolium* [26].

In this study, we aimed to screen the functional genes involved in triterpenoid saponin biosynthesis in *H*. *helix* using *de novo* transcriptome sequencing. Since hederacoside C, hederacoside B, hederacoside D and α-hederin are rich in leaves and hardly detectable in roots (S1 Fig), the transcriptomes between leaves and roots were compared to dissect the molecular basis of this tissue-specific accumulation. Interestingly, 2 pathways and 410 unigenes related to triterpenoid saponins biosynthesis were discovered in the present study, which enriched the database of molecular information for *H*. *helix*.

## Materials and methods

### Plant materials

One-year-old *H*. *helix* were cultivated from scions, and scion woods were collected from the Hunan Research Institute of Vine Plant and the green house in National Center for Citrus Improvement, Hunan Agricultural University, Hunan Province, China on April 11, 2104. Leaf and root tissues were collected randomly from these 1-year-old plants for transcriptome analysis on May 15, 2015. Tissues were rinsed in water, cut into small pieces, frozen in liquid nitrogen immediately and stored at −80°C for further analyses.

### RNA extraction, cDNA library construction and transcriptome sequencing

Leaf and root samples were harvested from five plants for RNA extraction and three biological replicates (L1 and R1, L2 and R2, L3 and R3) were performed. First, total RNA was extracted using RNA plant Plus Reagent (Tiangen, Beijing, China) according to the manufacturer’s instructions for polysaccharides & polyphenolics-rich samples. The total RNA purity was checked using a NanoDrop^®^2000 (Thermo, CA, USA) and the total RNA concentration and integrity were assessed using the RNA Nano 6000 Assay Kit of the Agilent Bioanalyzer 2100 system (Agilent Technologies, CA, USA).

The cDNA libraries were generated using the NEB Next^®^ Ultra^™^ RNA Library Prep Kit for Illumina^®^ (NE, USA) following the manufacturer’s recommendations, and index codes were added to attribute sequences to each sample. Poly-(A) mRNA was isolated from total RNA using Oligo-(dT) magnetic beads and fragmented in fragmentation buffer. First-strand cDNA was synthesized using a random hexamer primer and M-MuLV Reverse Transcriptase (RNase H-). Second-strand cDNA was synthesized using buffer, dNTPs, RNaseH, and DNA polymerase I. The remaining overhangs were converted into blunt ends via exonuclease/polymerase activities. After adenylation of the 3’ ends of DNA fragments, NEBNext Adaptor with a hairpin loop structure was ligated to prepare for hybridization. To select cDNA fragments preferentially 250–300 bp in length, the library fragments were purified with the AMPure XP system (Beckman Coulter, Beverly, USA). Then, 3 μL of USER Enzyme (NEB, USA) was used with size-selected, adaptor-ligated cDNA at 37°C for 15 min. PCR was performed with Q5 Hot Start HiFi DNA polymerase, Universal PCR primers and Index (X) Primer. PCR products were purified (AMPure XP system) and library quality was assessed on an Agilent Bioanalyzer 2100 system.

Clustering of the index-coded samples was performed using a cBot Cluster Generation System using the TruSeq PE Cluster Kit v4-cBot-HS (Illumina) according to the manufacturer’s instructions. After cluster generation, the library preparations were sequenced on an Illumina Hiseq 2500 platform, and paired-end reads were generated.

### De novo assembly, unigene annotation and functional classification

Raw reads obtained from HiSeq-2500 sequencing were filtered to exclude reads containing adaptors, reads with more than 5% unknown nucleotides, and low-quality reads. At the same time, the Q20, Q30 and GC-content of the clean data were calculated. All downstream analyses were based on clean data of high quality. *De novo* assembly of the transcriptome was performed with Trinity [27]. After assembly of clean reads with Trinity and removal of redundant sequences using the TGI Clustering Tool (TGICL) [28], clusters (prefixed with CL) and singletons (prefixed with Unigene) were finally obtained.

All assembled unique gene sequences were aligned against the non-redundant (Nr) protein database (http://www.ncbi.nlm.nih.gov/), SwissProt (http://www.expasy.ch/sprot/), Kyoto Encyclopedia of Genes and Genomes (KEGG) [29] (http://www.genome.jp/kegg/) and Clusters of Orthologous Groups (COG) (http://www.ncbi.nlm.nih.gov/cog/) databases using BLASTx algorithms with a threshold of E<1.0E-5, and the protein functional annotation information was searched against the Nt database using BLASTn algorithms with a threshold of E-value<0.00001 [30]. Gene ontology (GO) (http://www.geneontology.org) terms functional annotation was performed based on the best hits from Nr annotation using Blast2go software (http://www.blast2go.de/), and WEGO (http://wego.genomics.org.cn/cgi-bin/wego/index.pl) software was used for further resultant GO ID and classification [31,32]. All assembled unigenes were searched using the Gene ID listed in S1 Table.

### Differentially expressed unigene analysis

The expression levels of unigenes were established by in-house Perl scripts for each leaf and root sample; clean data were mapped back onto the assembled transcriptome and read-counts for each gene were obtained from the mapping results. To calculate the gene expression levels, the Fragments Per kb per Million fragments (FPKM) method was used [33]. The expression difference was identified by Perl scripts, in which Fisher’s exact test and the likelihood ratio were proposed to identify differentially expressed genes, and the P-value and false discovery rate (FDR) for each gene were calculated. Differentially expressed genes were required to have thresholds of “log2 ratio≥1” and “FDR<0.001” [34]. Next, GO and KEGG analysis were again performed on the DEGs.

### Quantitative real-time PCR analysis and RACE clone

The total RNA for RT-qPCR analysis was extracted using a RNAprep Pure Kit (Tiangen, Beijing, China), and 1.0 μg RNA was used for reverse transcription with a Fast Quant RT Kit (Tiangen, Beijing, China) in a 20-mL reaction volume according to the manufacturer's instructions. Gene-specific primer pairs (S2 and S3 Tables) were designed with Beacon Designer 8 software based on transcriptome-assembled data and synthesized by a commercial supplier (Sangon, Shanghai, China). The F-box gene was used as an internal control [35]. RT-qPCR was performed in 96-well plates in a Bio-Rad CFX96 real-time PCR system (Bio-Rad, CA, USA) with a SYBR Green-based PCR assay. The final volume for each reaction was 20 mL with the following components: 2 mL diluted cDNA template (1 mg/mL), 10 mL SYBR Green Mix (Bio-Rad, CA, USA), 2.5 mL forward primer (2.5 mM), 2.5 mL reverse primer (2.5 mM) and 3 mL ddH_2_O. The reaction was conducted under the following conditions: 95°C for 3 min, followed by 40 cycles of denaturation at 95°C for 10 s and annealing/extension at 56°C for 30 s. The melting curve was obtained by heating the amplicon from 65°C to 95°C at increments of 0.5°C per 5 s. Each RT-qPCR analysis was performed with three biological replicates. The relative quantification of gene expression was computed using the 2^−ΔΔCt^ method. The full-length cDNAs of the two genes were cloned by RACE with a SMARTer RACE 5’/3’ Amplification kit (Clontech, CA, USA) according to the user manual. The RACE fragments were amplified by specific primers, ligated into the pRACE vector (Clontech, CA, USA) and sequenced. Then, the RACE fragments were used for the subsequent verification and analysis of full-length cDNAs.

## Results

### Reads generation and De novo assembly

**Fig 1:**
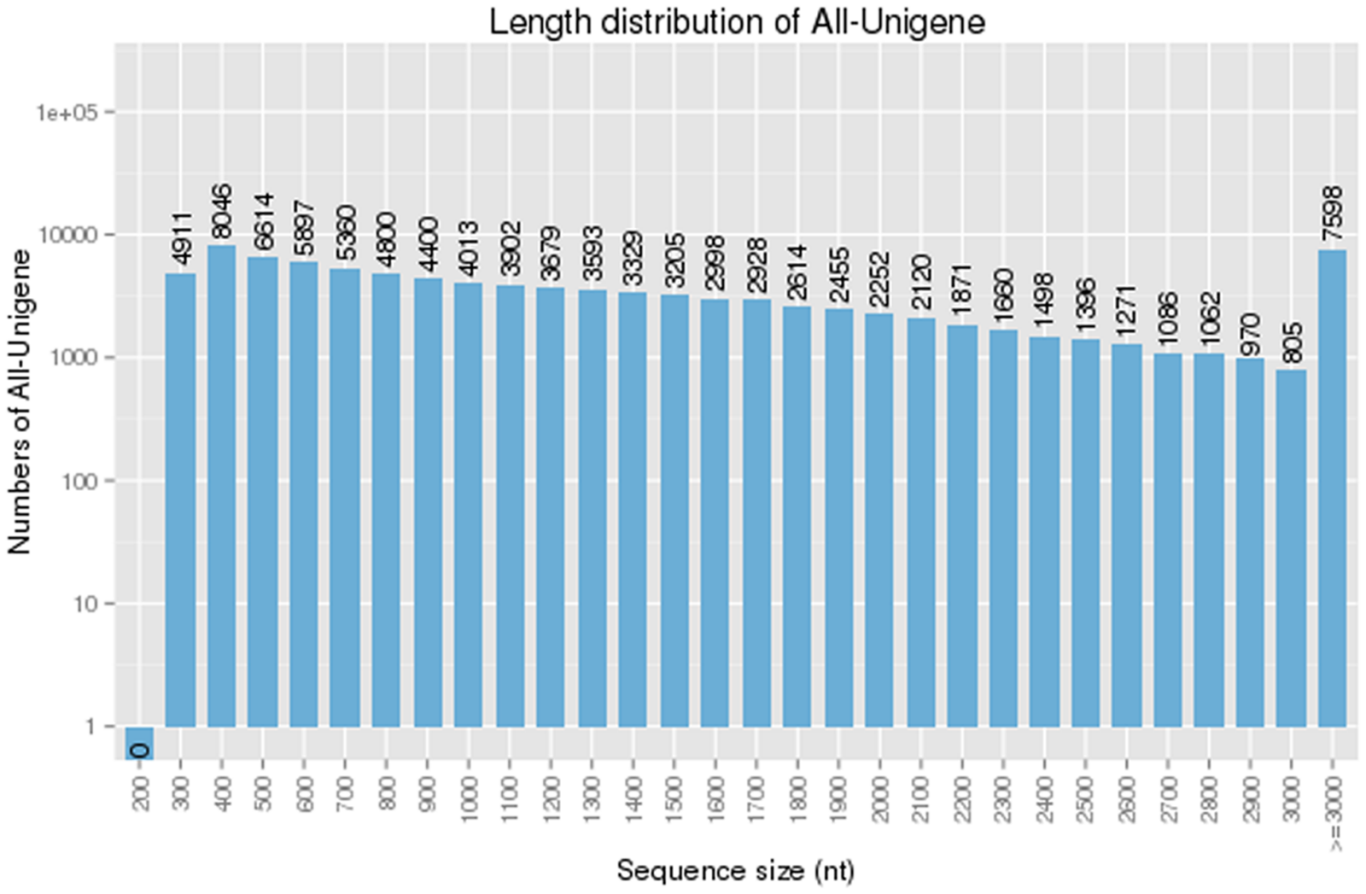
Length distribution frequency of unigenes in *H*. *helix*.

Six cDNA libraries prepared from three leaves and three roots tissue of *H*. *helix* were sequenced using the Illumina HiSeq^™^2500 platform. A total of 138,709,902 and 139,062,838 raw reads were generated, and after removing adapters and filtering the low-quality sequences, 123,162,610 and 122,589,364 clean reads were generated for the leaf (L1, L2, L3) and root (R1, R2, R3) libraries (Table 1). Since there is no reference genome sequence in *H*. *helix*, all clean reads from these six libraries were *de novo* assembled into contigs using the Trinity software, and reads were mapped back to contigs, redundancy was removed and the reads were assembled further using TGICL [28]. Finally, 96,333 unigenes were obtained with a mean length of 1385 nucleotides (nt) (N50 1927 nt). A detailed summary of the sequencing and assembly results is shown in Table 2 and the length distribution of all unigenes is shown in Fig 1.

**Table 1:** Summary of data output quality of various libraries.

Library	Raw Reads	Clean Reads	Error (%)	Q20 (%)	Q30 (%)	GC (%)
L1	45,630,708	39,909,334	0.1	97.08	95.40	45.05
L2	45,480,900	40,761,120	0.1	97.14	95.71	44.72
L3	47,598,294	42,492,156	0.1	97.10	95.46	44.89
R1	46,941,114	40,930,938	0.1	96.65	95.76	44.73
R2	46,855,462	41,306,100	0.1	97.16	95.52	44.54
R3	45,266,262	40,352,326	0.1	97.10	95.68	44.58

**Table 2:** Summary of assembly results of *H*. *helix*.

Samples	Total Number	Total Length (nt)	Mean Length (nt)	N50 (nt)	Distinct Clusters	Distinct Singletons
L1-Contig	116,550	99,922,035	857	1339	-	-
L2-Contig	117,656	101,212,619	860	1341	-	-
L3-Contig	114,891	97,770,836	851	1323	-	-
R1-Contig	136,187	111,354,934	818	1271	-	-
R2-Contig	140,439	115,262,101	821	1280	-	-
R3-Contig	128,613	104,187,419	810	1267	-	-
L1-Unigene	71,734	69,222,524	965	1511	34,970	36,764
L2-Unigene	70,985	69,495,387	979	1526	35,544	35,441
L3-Unigene	68,637	66,692,896	972	1514	34,305	34,332
R1-Unigene	81,825	75,839,021	927	1462	38,521	43,304
R2-Unigene	85,302	79,392,368	931	1474	39,032	46,270
R3-Unigene	78,940	71,597,869	907	1440	35,493	43,447
All-Unigene	96,333	133,417,819	1385	1927	55,721	40,612

### Functional annotation and classification

**Fig 2:**
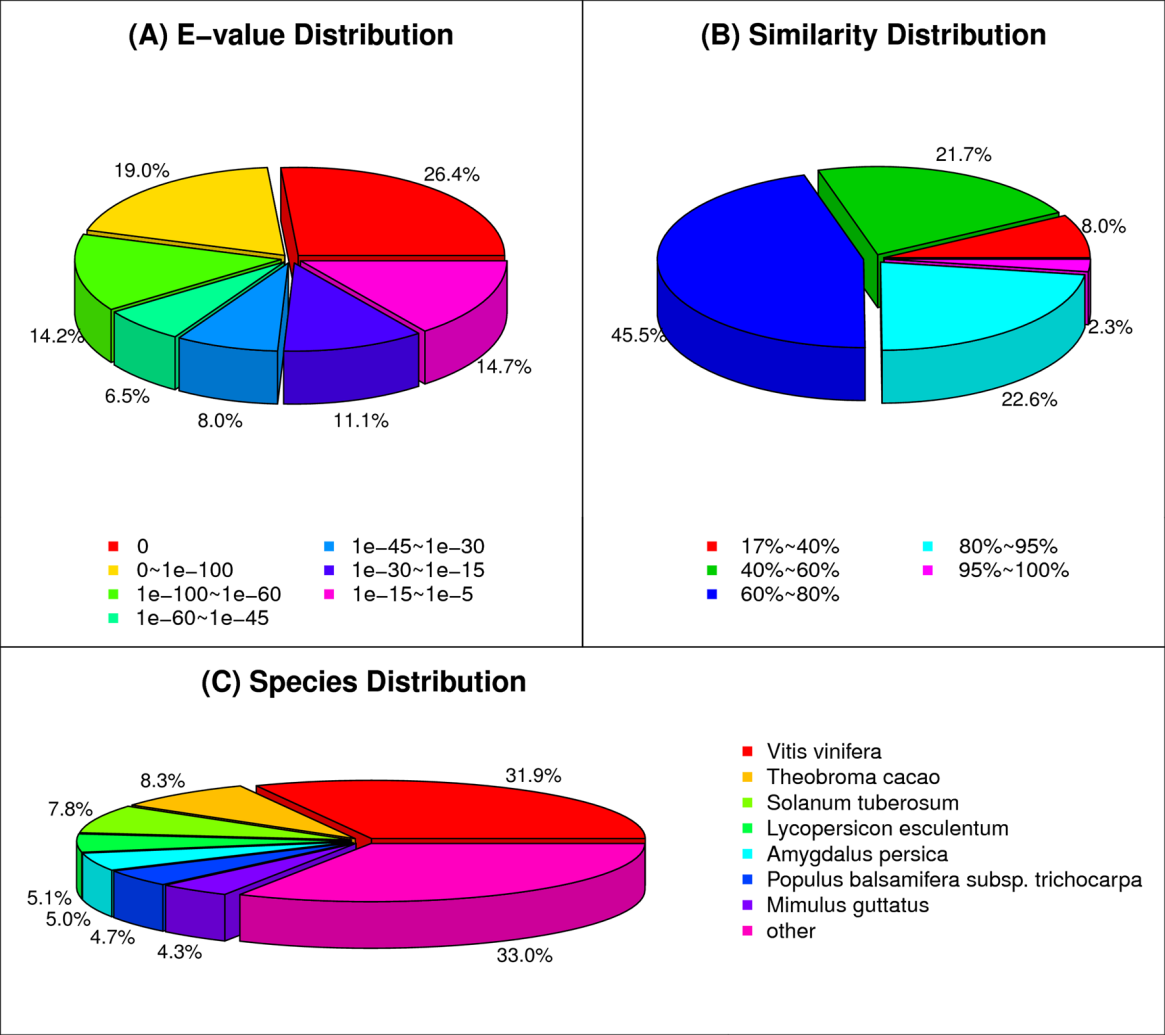
Gene similarity of unigenes against the Nr database. (A) E-value distribution of top BLAST hits for each unigene (E-value of 1.0E-5). (B) Similarity distribution of best BLAST hits for each unigene. (C) Distribution of BLAST results by species shown as percentage of total homologous sequences (E-value ≤1.0E-5). All plant proteins in the NCBI Nr database were used for homology search and the best hit of each sequence was used for analysis.

All assembled unigenes were first searched against the Nr protein, Swissprot protein, KEGG, GO and COG databases using the Blastx program with E-value threshold of 1E-5. Of the 96,333 unique sequences, 79,085 unigenes (82.1%) had at least one significant match to an existing gene model. The detailed results are shown in Table 3 and S1 Table. Based on the Nr database, the E-value distribution showed that approximately 54.5% of the mapped unigenes ranged from 1E-5 to 1E-100 (Fig 2A). As shown in the similarity distribution, approximately 24.9% unigene sequences have a similarity above 80%, while 67.2% of the hits have a similarity from 40% to 80% (Fig 2B). Furthermore, 31.9% of the *H*. *helix* unigenes sequences are homologous to genes of *Vitis vinifera*; the others are 8.3% to *Theobroma cacao*, 7.8% to *Solanum tuberosum*, 5.1% to *Lycopersicon esculentum*, 5.0% to *Amygdalus persica*, 4.7% to *Populus balsamifera* subsp. trichocarpa and 4.3% to *Mimulus guttatus*, respectively (Fig 2C).

**Table 3:** Summary of functional annotations of *H*. *helix* unigenes.

Public database	Number of Unigenes	Percentage (%)
Annotated in Nr	75,773	78.7
Annotated in Nt	70,728	73.4
Annotated in SwissProt	51,320	53.3
Annotated in KEGG	47,100	48.9
Annotated in COG	32,443	33.7
Annotated in GO	50,479	52.4
All annotated Unigenes	79,085	82.1
Total Unigenes	96,333	100

**Fig 3:**
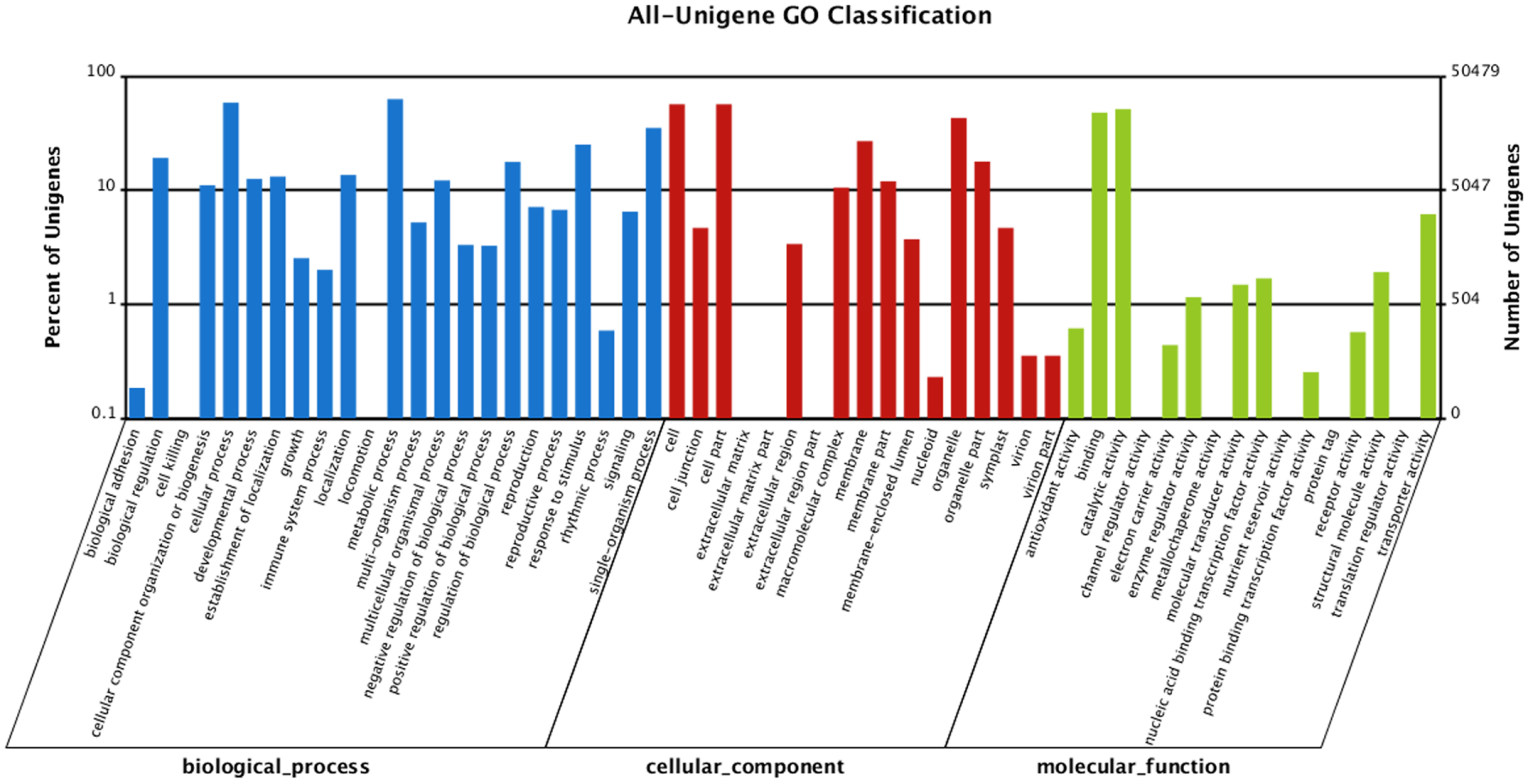
Comparison of Gene ontology (GO) classifications of *H*. *helix*. Results are summarized into three main GO categories (biological process, cellular component, molecular function) and 44 sub-categories. The x-axis indicates subcategories; right y-axis indicates number of genes in a category; and left y-axis indicates percentage of a specific category of genes in the main category.

GO analysis is an international standard system of gene function classification, that has three main ontologies to describe molecular functions, cellular components and biological processes. Based on the Nr annotation, we used GO analysis to classify functions and understand the general distribution of the unigenes of *H*. *helix*. Among the 75,773 annotated unigenes, 50,479 sequences were categorized into 56 GO functional groups, as shown in Fig 3 and S4 Table. The biological process category has the highest number of unigenes among the three categories, followed by the cellular components category and molecular function category. From the overall analysis, there are 15 items clustering more than 5,047 unigenes. Within the biological processes category, “cellular process” and “metabolic process” have the highest number of unigenes. Meanwhile, “cell”, “cell part” and “organelle” were enriched in the cellular components category. Genes encoding “binding” proteins and proteins related to “catalytic activity” were the largest proportion in the molecular function category.

**Fig 4:**
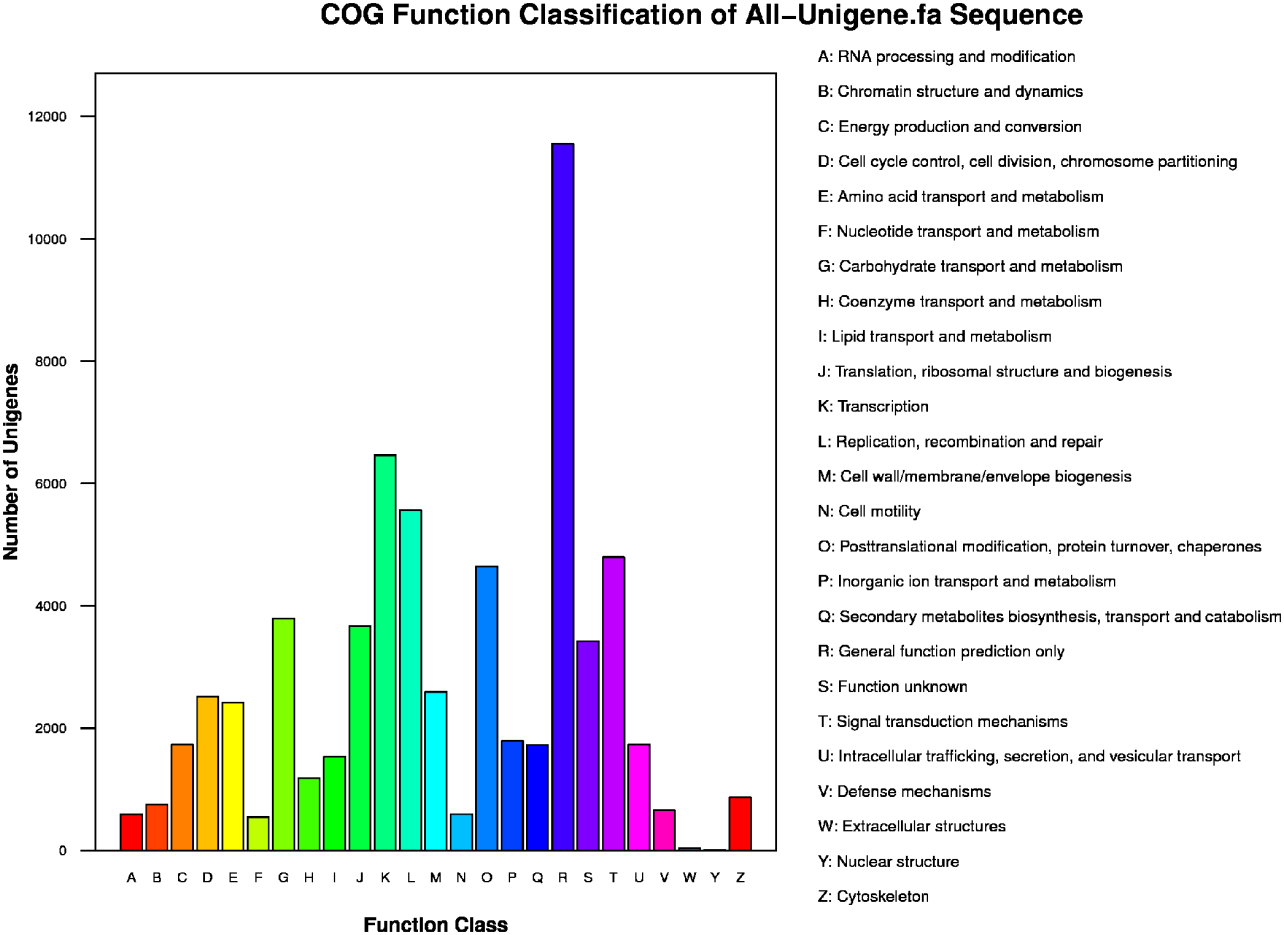
COG function classification of *H*. *helix* transcriptome. A total of 33,205 unigenes showed significant homology (E-value ≤1.0E-5) to genes in one of the 25 categories (A-W, Y and Z) in the NCBI COGs database.

Using COG classification to further evaluate the completeness and effectiveness of the *H*. *helix* annotation, a total of 32,443 annotated sequences were assigned to 25 COG categories. As shown in Fig 4 and S5 Table, the cluster of “General function prediction” represents the largest group (11,547, 35.6%), followed by “Transcription” (6,459, 19.9%), “Replication, recombination and repair” (5,560, 17.1%), “Signal transduction mechanisms” (4,791 14.8%) and “Posttranslational modification, protein turnover, chaperones” (4,643 14.3%). The smallest group was “Nuclear structure”, with only 8 sequences.

For KEGG analysis, a total of 96,333 annotated unigenes were mapped to identify active pathways in *H*. *helix* with a cut-off of E-value<0.00001. Of these, 47,100 with significant matches from the database were assigned to 128 KEGG pathways (S6 Table). The largest category was metabolic pathways (10,484, 22.3%), followed by biosynthesis of secondary metabolites (4,795, 10.2%), plant-pathogen interactions (2,805, 6.0%), plant hormone signal transduction (2,623, 5.6%) and spliceosome (2,074, 4.4%). These annotations provide a further understanding of the transcriptome data and their functions and pathways in *H*. *helix*.

## Differentially Expressed Gene analysis

**Fig 5:**
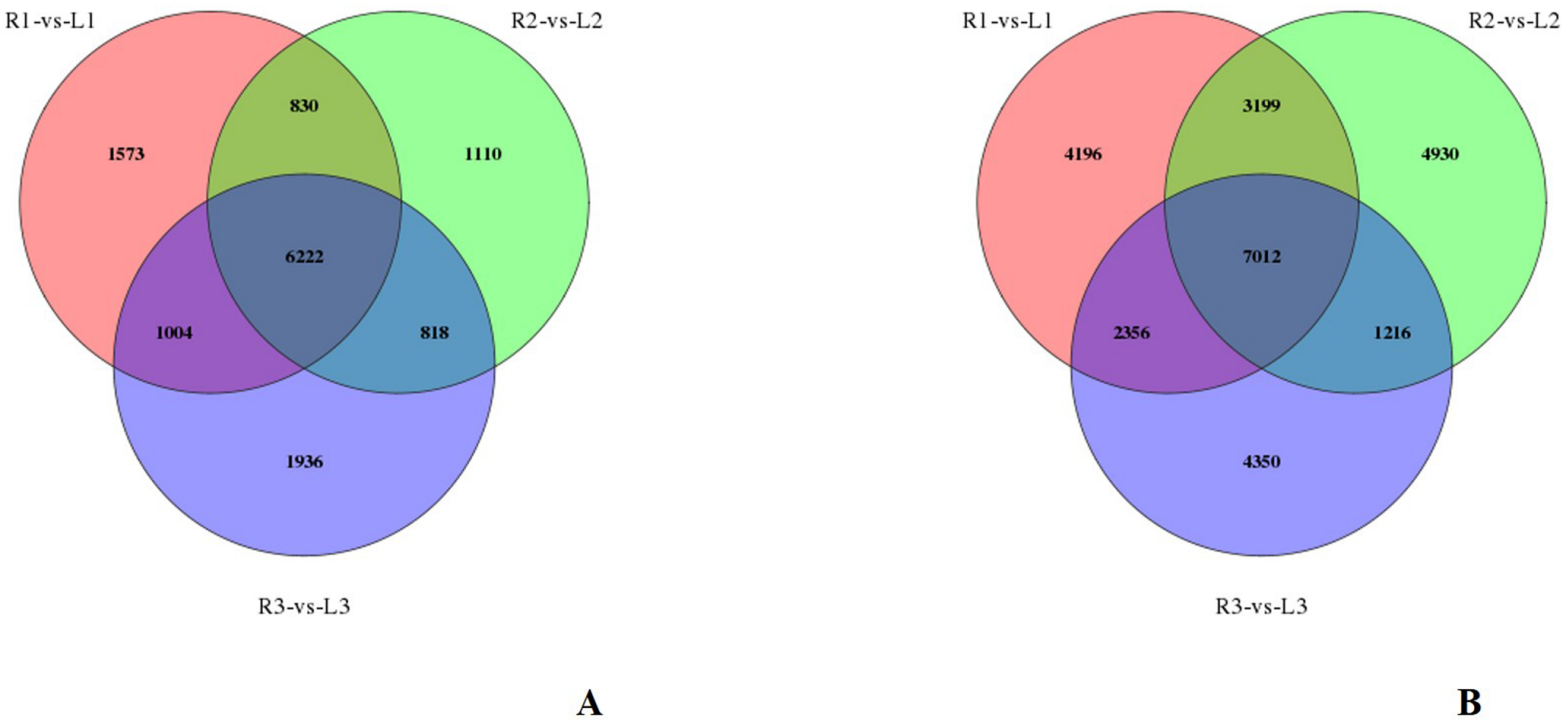
Differentially expressed gene analysis of six libraries in *H*. *helix*. (A) Expressed higher unigenes in leaf samples. (B) Expressed lower unigenes in leaf samples.

The Differentially Expressed Genes (DEG) of the six transcriptome libraries were used to discover the unigenes with significant differences in expression. In this study, the expression of unigenes was calculated by FPKM. The different analysis methods were as follows: R1 and L1, R2 and L2, R3 and L3, were compared, and the DEGs found were used to comment on all three replicates for GO classification and KEGG pathway analysis. In total, 6,222 unigenes were more expressed higher and 7,012 unigenes were expressed lower in leaf samples than root samples (S7 and S8 Tables). According to the expression differences between the roots (R) and Leaves (L) shown in Fig 5, GO and KEGG analysis were performed again based on the Nr annotation. Of the 13,234 DEGs, 8,771 were assigned to 52 GO categories, and of these unigenes, 4,243 of the 6,222 expressed higher unigenes and 4,528 of the 7,012 down-expressed lower unigenes were assigned to at least one of the GO terms (biological process, cellular component, or molecular function). After KEGG analysis was performed again, 3,657 of the 6,222 expressed higher unigenes were annotated into 123 pathways and 4,324 of the 7,012 expressed lower unigenes were annotated into 122 pathways. Meanwhile, the top 20 statistically concentrated pathways were analysed based on the false discovery rate (FDR)≤0.001 and these results can identify the main biochemical metabolic pathways and signal transduction pathways that the DEGs attended, as seen in S2 Fig.

### Identification of triterpene saponin biosynthesis genes

In addition to the KEGG analysis and unigenes functional annotation results, several triterpene saponin biosynthesis genes were discovered and identified. In the KEGG analysis results of 96,333 unigenes, two pathways related to triterpene saponin biosynthesis in *Hedera helix* L., i.e., the terpenoid backbone biosynthesis and sesquiterpenoid and triterpenoid biosynthesis pathways, were assigned; these two pathways contained the MVA and MEP pathways upstream and carbocyclic biosynthesis midstream. From the annotation results, 339 unigenes were mapped to the terpenoid backbone biosynthesis pathway and 71 unigenes were mapped to the sesquiterpenoid and triterpenoid biosynthesis pathway. Among these unigenes, the genes encoding key enzymes involved in triterpene saponin biosynthesis are listed in full in Table 4: 6 putative genes (*AACT*, *HMGS*, *HMGR*, *MVK*, *PMVK*, *MVD*) and 8 putative genes (*DXS*, *DXR*, *ispD*, *ispE*, *ispF*, *ispG*, *ispH*, *IDI*) were discovered in the MVA and MEP pathway, respectively, and 6 putative genes (*GPS*, *GGPS*, *FPS*, *SS*, *SE*, *β-AS*) were discovered for carbocyclic biosynthesis. In most cases, more than one unigene can be annotated to the same enzyme encoded gene or gene family [36,37]; this phenomenon was observed in this study. As shown in Table 4, most genes had multiple alleles or paralogues in the transcriptome, such as *1-deoxy-D-xylulose-5-phosphate synthase* (*DXS*, EC: 2.2.1.7), *geranylgeranyl diphosphate synthase* (*GGPS*, EC: 2.5.1.29) and *squalene synthase* (*SS*, EC: 2.5.1.21), these genes were annotated with nine paralogues. Meanwhile, in the above pathways related to triterpene saponin biosynthesis, we also identified genes with different expression levels in leaf compared to roots using combined DEG analysis and FPKM calculation results. There are 13 unigenes belonging to 8 putative genes expressed higher and 8 unigenes belonging to 4 putative genes expressed lower in leaves (Table 4). In addition, the modification of the triterpenoid carbocyclic is a putative downstream pathway of triterpene saponin biosynthesis in *H*. *helix*; specifically, CYP450s and GTs played a major role in this part. In this study, a total of 269 and 197 unigenes were identified for the CYP450 family and GT family, respectively (S9 and S10 Tables).

**Table 4:** Discovery and expression of unigenes involved in triterpenesaponin biosynthesis in *Hedera helix L*.

Enzymes name	Abbreviation	EC number	Putative ortholog	Expressed higher	Expressed lower
Acetyl-CoA acetyl transferase	AACT	EC:2.3.1.9	CL10734, CL8643		
Hydroxymethylglutaryl CoA synthase	HMGS	EC:2.3.3.10	CL4883, Unigene7122		
3-hydroxy-3-methylglutaryl-coenzyme A reductase	HMGR	EC:1.1.1.34	CL84, Unigene12643, Unigene12948, Unigene9993,	Unigene9993	CL84, Unigene12643
Mevalonate kinase	MVK	EC:2.7.1.36	CL10135, Unigene31942, Unigene37001		CL10135
Phosphomevalonate kinase	PMVK	EC:2.7.4.2	CL2755		
Mevalonate diphosphosphate decarboxylase	MVD	EC:4.1.1.33	CL12343		
1-deoxy-D-xylulose-5-phosphate synthase	DXS	EC:2.2.1.7	CL1741, CL6964, CL7506, Unigene12956, Unigene20321, Unigene26465, Unigene26467, Unigene26469, Unigene7001	CL1741	
1-deoxy-D-xylulose-5-phosphate reductoisomerase	DXR	EC:1.1.1.267	CL4453, Unigene20397, Unigene27513, Unigene32536, Unigene5694		CL4453, Unigene5694
2-C-methyl-D-erythritol 4-phosphate cytidylyltransferase	ispD	EC:2.7.7.60	CL177, Unigene28885		
4-diphosphocytidyl-2-C-methyl-D-erythritol kinase	ispE	EC:2.7.1.148	Unigene15885, Unigene29532		
2-C-methyl-D-erythritol 2,4-cyclodiphosphate synthase	ispF	EC:4.6.1.12	Unigene5248	Unigene5248	
(E)-4-hydroxy-3-methylbut-2-enyl-diphosphate synthase	ispG	EC:1.17.7.1	CL4323, Unigene18905, Unigene21207, Unigene39208		
4-hydroxy-3-methylbut-2-enyl diphosphate reductase	ispH	EC:1.17.7.4	CL3923, Unigene11112, Unigene39239, Unigene40179	CL3923	
Isopentenyl-diphosphate Delta-isomerase	IDI	EC:5.3.3.2	Unigene9222		
Geranyl diphosphate synthase	GPS	EC:2.5.1.1	CL3895, Unigene23216, Unigene31228		
Geranylgeranyl diphosphate synthase	GGPS	EC:2.5.1.29	CL12915, CL1497, CL3631, CL5060, CL8765, Unigene20086, Unigene26473, Unigene6117, Unigene7971		Unigene26473, CL1497, Unigene20086
Farnesyl diphosphate synthase	FPS	EC:2.5.1.10	CL8585, Unigene7386, Unigene7389, Unigene256	CL8585	
Squalene synthase	SS	EC:2.5.1.21	CL11265, Unigene15662, Unigene17742, Unigene18488, Unigene28187, Unigene30735, Unigene35625, Unigene7507, Unigene14696	CL11265, Unigene35625, Unigene7507, Unigene15662	
Squaleneepoxidase	SE	EC:1.14.14.17	CL6504, CL9719, CL9981, Unigene14156, Unigene17245	CL6504,CL9981	
β-amyrin synthase	β-AS	EC:5.4.99.39	CL5897, CL1580, CL11721, Unigene29516, Unigene32344	CL11721, CL1580	

## Validation by RT-qPCR and RACE Clone

**Fig 6:**
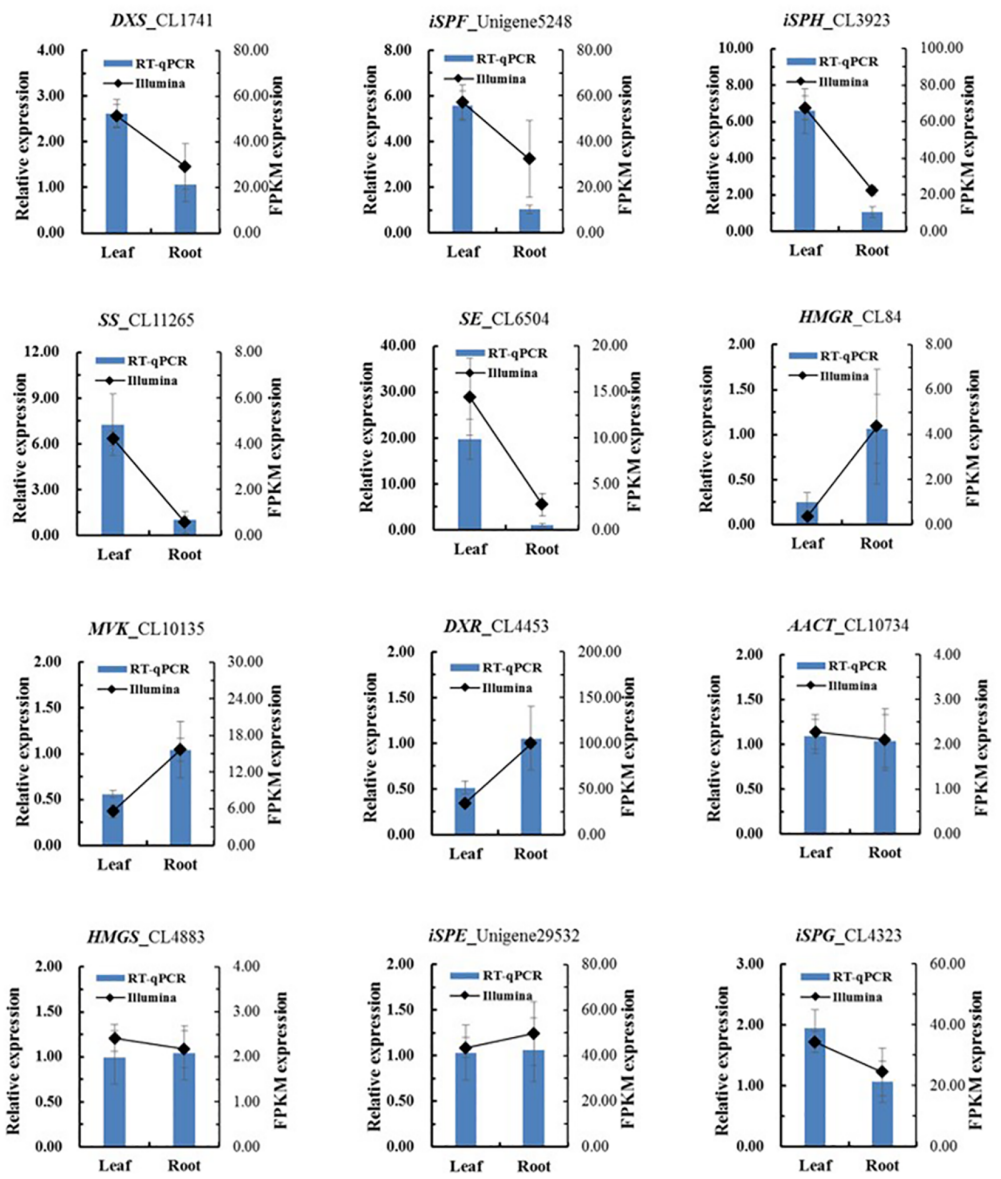
RT-qPCR validation of selected unigenes involved in triterpene saponin biosynthesis. Columns indicate relative expression obtained by RT-qPCR (left y-axis); lines indicating the expression level were calculated by FPKM method (right y-axis). All data are presented as mean value of three repeats.

To confirm the accuracy of the Illumina paired-end sequencing and FPKM calculated results, we selected 12 unigenes and used RT-qPCR to determine their relative expression level in the leaf and root tissues of *H*. *helix*. All 12 unigenes were triterpene saponin biosynthesis genes and contained 5 higher-expressed unigenes unigenes (*DXS*_CL1741, *iSPF*_Unigene5248, *iSPH*_CL3923, *SS*_CL11265, *SE*_CL6504), 3 lower-expressed unigenes unigenes (*HMGR*_CL84, *MVK*_CL10135, *DXR*_CL4453) and 4 unchanged unigenes (*AACT*_CL10734, *HMGS*_CL4843, *iSPE*_Unigene29532) as calculated by FPKM. The RT-qPCR and FPKM results were compared and are presented in Fig 6, the expression levels are similar.

To further validate the *de novo* assembly results, we also cloned the full-length cDNA of 2 genes (*HMGR accession No*. KX056076, *SE accession No*. KU942524) from the *H*. *helix* leaf using the RACE method according to fragments of the transcriptome (*HMGR*_CL84, *SE*_CL6504). The full-length cDNA sequence of *HMGR* is 2215 bp and includes a complete open reading frame of 1731 bp, encoding a polypeptide of 577 amino acids (S3 Fig). A phylogenetic tree shows that the HMGR gene and SE gene in *H*. *helix* are highly homologous with other species. The predicted molecular weight of the HMGR putative protein is 61.98kD, with a theoretical pI of 7.52. Similarly, the full-length cDNA sequence of *SE* is 2040 bp and includes a complete open reading frame of 1615 bp, encoding a polypeptide of 537 amino acids (S4 Fig). A phylogenetic tree was built to investigate the evolutionary relationship with other plants and found higher homology between them. The putative protein of SE had a 58.69-kD predicted molecular weight with a theoretical pI of 8.95. Both the full-length cDNA sequences of *HMGR* and *SE* had a Poly (A) signal sequence.

## Discussion

As it is a traditional medicinal plant in Europe, previous studies of *H*. *helix* have mainly focused on its pharmacology, efficacy and active components [1,3,7]; molecular biology research is less common, and genomic and transcriptomic data are still not available in NCBI database for this plant. As the first study using next-generation sequencing technology to obtain transcript coverage of *H*. *helix*, our deep sequencing provides important, fundamental molecular data. In the current study, the clean reads and *de novo* assembled unigenes were obtained using the Illumina HiseqTM 2500 platform (3 replicates of leaf and root tissues, 6 libraries in total). Their N50 length and average length indicated that all sequencing results were assembled effectively and with high quality [37,38]. In transcriptome analysis without a reference genome, unigenes were mapped in a public database, and unigenes with a high matching rate by the homology principle were annotated and classified. The results indicate that the transcriptomes of *H*. *helix* have great accuracy and integrity and can be used for functional genes studies or other molecular biology studies on *H*. *helix*.

As triterpenoid saponins are the main functional components in *H*. *helix* [4,9,10], here we aimed to expand knowledge of their biosynthesis pathway and screen related functional genes using transcriptome sequencing. The HPLC results clearly show that triterpenoid saponin contents are significantly different in leaves and roots (S1 Fig); therefore, choosing the leaf and root for comparative transcriptome analysis will greatly facilitate dissection of the genes involved in organ-specific secondary metabolite biosynthesis of triterpenoid saponins. This approach is widely used for mining and identifying novel genes in secondary metabolite biosynthesis [22, 39]. In this study, with *de novo* assembling and deciphering, the terpenoid biosynthesis backbone and sesquiterpenoid and triterpenoid biosynthesis pathway were addressed, and 20 functional genes were found. Although the two pathways are common pathways in the biosynthesis of terpenoids and sterols reported in *Panax notoginseng* [36] and *Solanum elaeagnifolium* [26], our results provide the first accurate and comprehensive gene information for *H*. *helix*.

CYP450s [19, 40, 41] and GTs [42,43], which modify the triterpene carbon ring, play key roles in the biosynthesis of various natural products. Moreover, CYP450s catalyse an irreversible oxygenating reaction and are multifunctional enzymes with a very complicated functional classification. Therefore, they have attracted special attention in metabolic engineering. However, in the synthesis pathway of terpenoid-based natural products, only 19 CYP450s gene functions have been validated [40].

Based on our transcriptome sequencing, we annotated 4 CYP450s genes in terpenoid biosynthesis: CYP716A52v2 with β-Amyrin 28-oxidase function, which produces oleanane-type ginsenosides [44]; CYP716A47 with protopanaxadiol synthase function that produces protopanaxadiol [45], CYP72A1 with secologanin synthase function that produces secologanin [46], CYP71D55 with premnaspirodiene oxygenase function that produces solavetivone [47]. Due to the versatile function of CYP450s, the exact function of the annotated CYP450s in terpenoid biosynthesis must still be illustrated. Glycosyltransferase (GT) participates in the last step in triterpenoid saponin biosynthesis; the GT family also has a complicated function as a member of the CYP450 family. Lahoucine Achine *et al* accurately validated that Ugt73k1 and Ugt71g1 participates in triterpene saponin biosynthesis [48]. In our transcriptome, there are 197 unigene with GT activity; however, we did not annotate these two GT genes but only annotated the obtained unigenes belonging to the Ugt73 sub-family. This indicates that GTs are not conserved in different species, even when catalysing the same reaction.

Testing and verification of the accuracy of *de novo* assembled sequences is one of the most crucial steps for no-reference transcriptome research. In this study, we calculated the relative expression of 12 unigenes using an RT-qPCR method and cloned full-length putative *HMGR* and *SE* genes for validation. These two-part results mutually confirmed that our *de novo* assembled sequences were accurate and useful. The twelve chosen unigenes all belong to the triterpenoid saponins biosynthesis pathway and represent three types of expression patterns (higher, lower, or no change) in leaf transcripts by FPKM calculation. *HMGR* and *SE* were selected as examples to validate the accuracy because they represent the genes with highest and lowest expression level in leaf samples. In addition, *HMGR* is the first rate-limiting enzyme in the terpenoid biosynthesis pathway, and many reports have presented *HMGR* as an important gene in the terpenoid biosynthesis pathway [49, 50]; *SE* participates in the first oxidation reaction in the triterpenoid saponins biosynthesis pathway and promoting squaleneep oxidation to produce 2,3-oxidosqualene. *SE* is also an important regulation gene, as indicated by many studies [51, 52]. In summary, this transcriptome is successful and reliable and can provide data to support further molecular research on *H*. *helix*.

## Conclusions

In the current study, comparative transcriptomes between leaf and root tissues in H. helix were implemented using the Illumina HiseqTM 2500 platform. After *de novo* assembly and sequence annotation, a total of 96,333 unigenes were obtained with a mean length of 1,385 nt; 32,443 annotated sequences were assigned to 25 COG categories, 50,479 sequences were categorized into 56 GO functional groups and 47,100 unigenes were assigned to 128 KEGG pathways. After differentially expressed gene analysis, 6,222 unigenes were more highly expressed and 7,012 unigenes were less expressed in leaf samples. The RT-qPCR analysis and RACE clone results indicated that *de novo* assembled sequences were accurate and valuable. Two pathways and 20 putative genes related to triterpenoid saponin biosynthesis were discovered. These data will enrich our knowledge and provide a theoretical foundation for molecular research on *H*. *helix*.

## Supporting information

S1 Fig HPLC chromatogram of different tissue samples of *H*. *helix*. (TIF)

S2 Fig Scatter plot of top 20 KEGG pathway enrichment for DEGs. (TIF)

S3 Fig The amino acid sequence of *HMGR* gene in *H*. *helix*. (TIF)

S4 Fig The amino acid sequence of *SE* gene in *H*. *helix*. (TIF)

S1 Table Annotation summary. (XLS)

S2 Table Primers for RT-qPCR analysis. (DOCX)

S3 Table Primer sequences for RACE clone. (DOCX)

S4 Table All-Unigene GO analysis. (XLS)

S5 Table All-Unigene COG classification. (XLS)

S6 Table All-Unigene KEGG analysis. (XLS)

S7 Table Expressed higher DEGs in leaf. (XLS)

S8 Table Expressed lower DEGs in leaf. (XLS)

S9 Table Annotation_CYP450s. (XLS)

S10 Table Annotation_GTs. (XLS)
