# Supplementary figures and images for: De novo leaf and root transcriptome analysis to identify putative genes involved in triterpenoid saponins biosynthesis in Hedera helix L
Source: PLoS One. 2017 Aug 3;12(8):e0182243. doi: 10.1371/journal.pone.0182243 (PMC5542655; doi:10.1371/journal.pone.0182243)

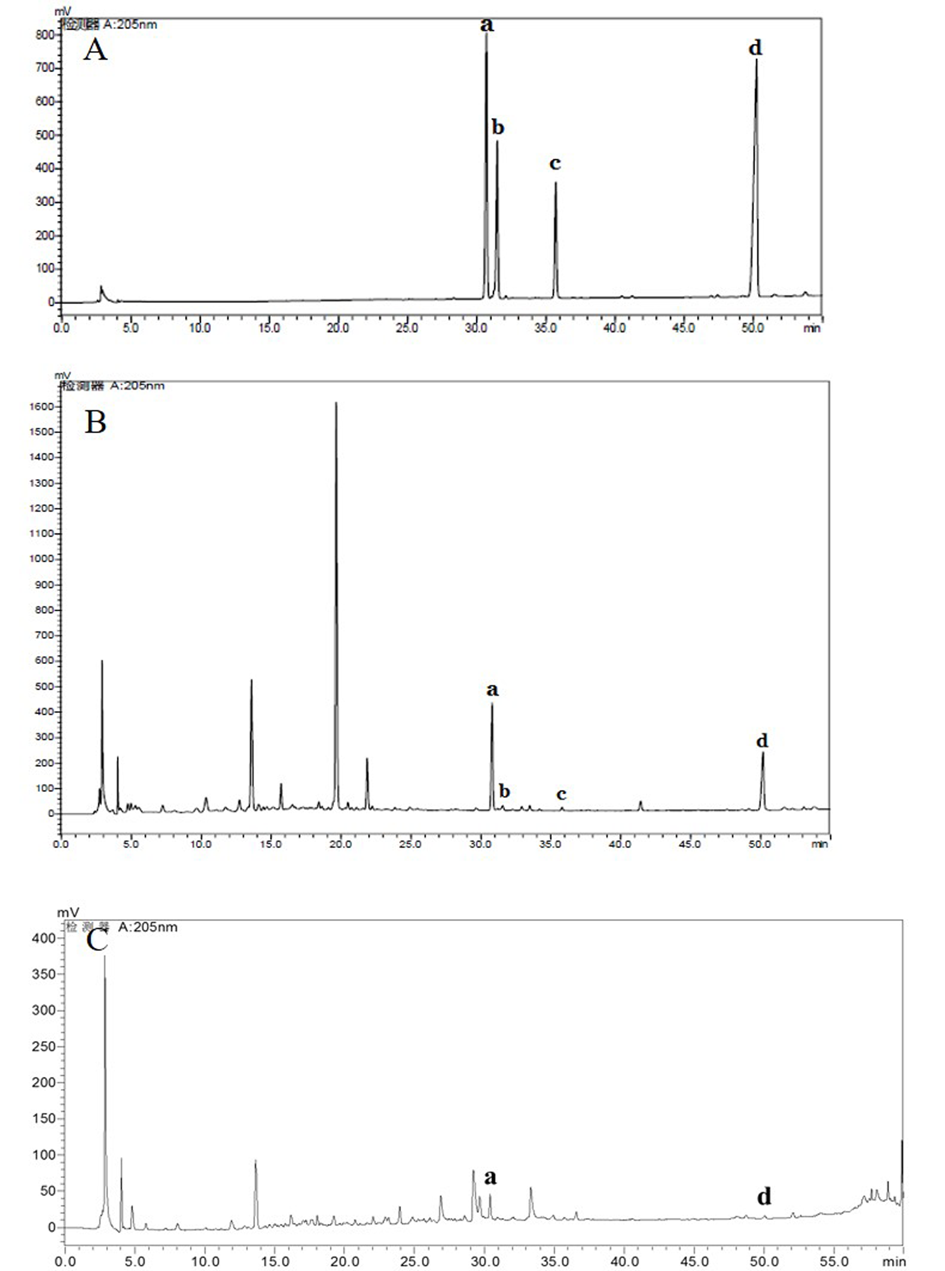

Supplement: S1 Fig — (TIF) [file pone.0182243.s001.tif]

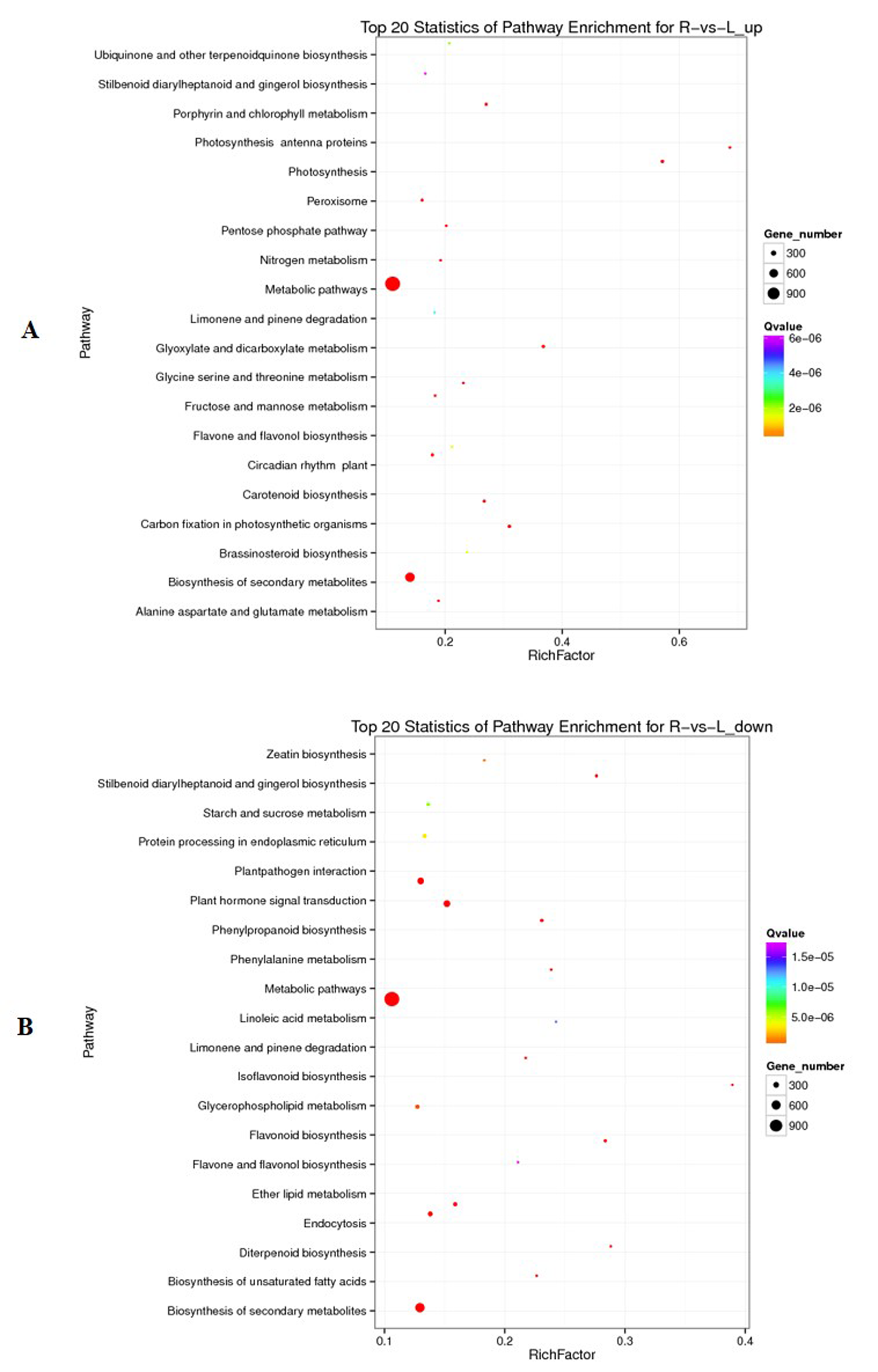

Supplement: S2 Fig — (TIF) [file pone.0182243.s002.tif]

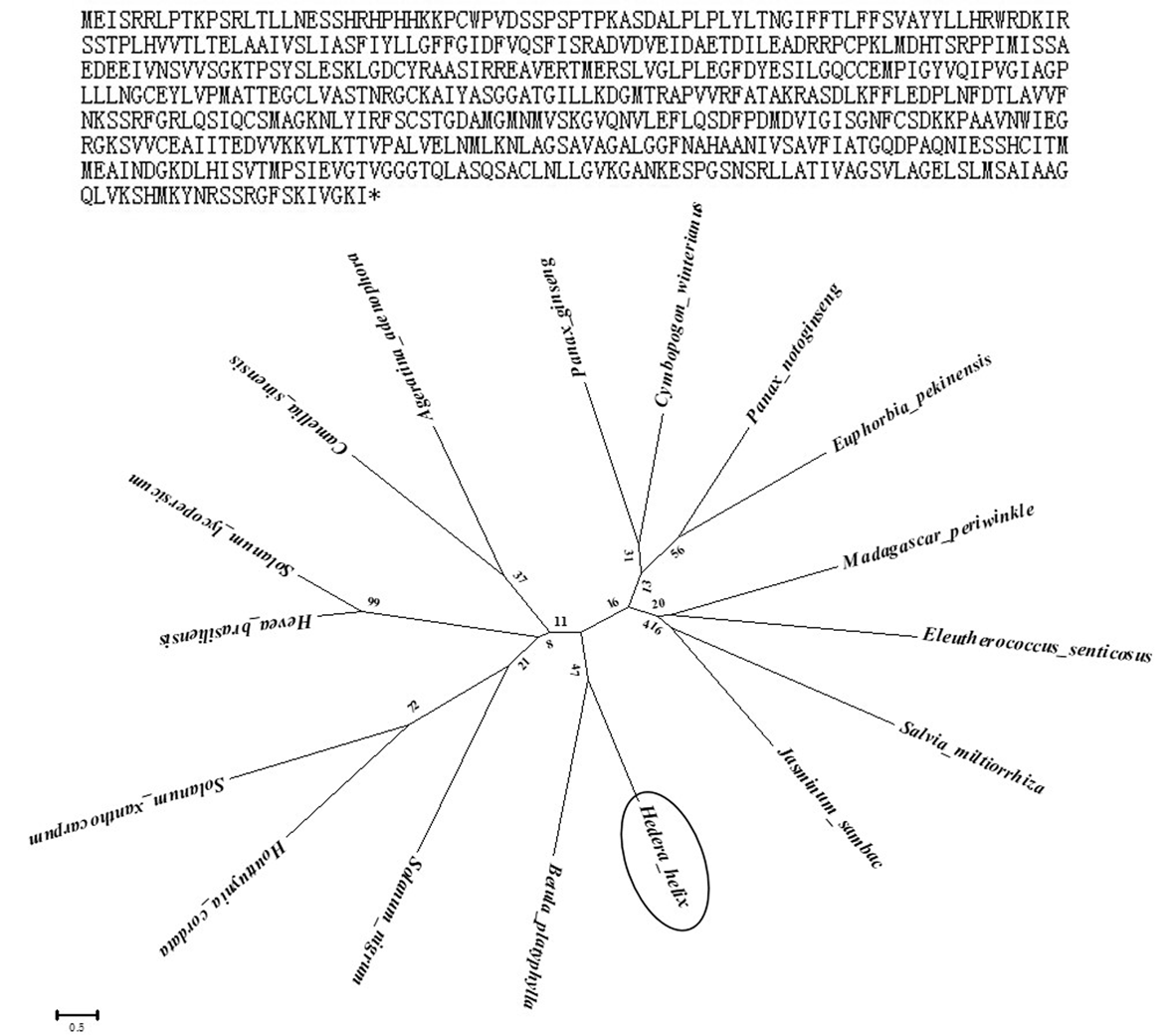

Supplement: S3 Fig — (TIF) [file pone.0182243.s003.tif]

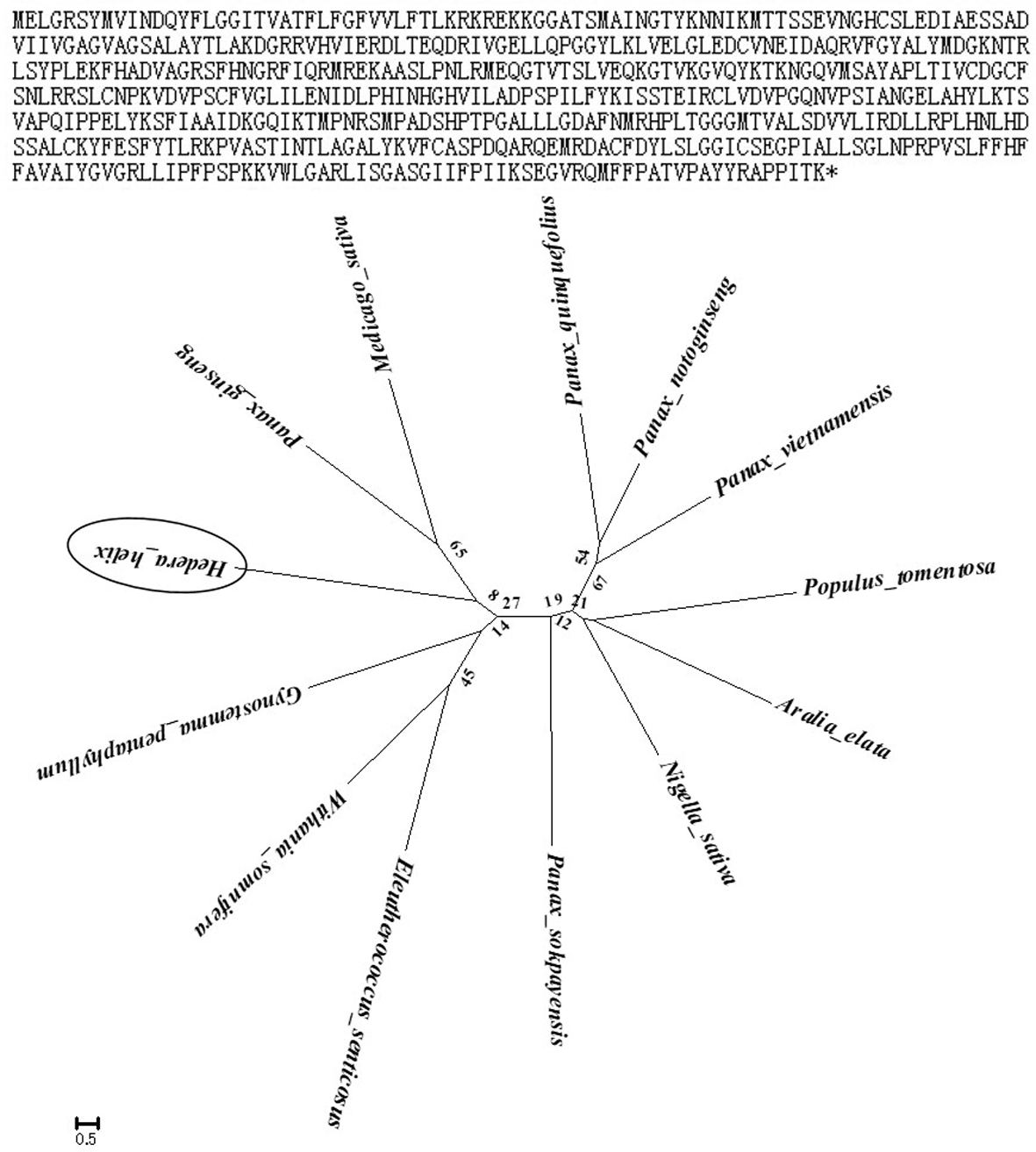

Supplement: S4 Fig — (TIF) [file pone.0182243.s004.tif]
