# Supplementary material for: De novo leaf and root transcriptome analysis to identify putative genes involved in triterpenoid saponins biosynthesis in Hedera helix L
Source: PLoS One. 2017 Aug 3;12(8):e0182243. doi: 10.1371/journal.pone.0182243 (PMC5542655; doi:10.1371/journal.pone.0182243)
Supplement: S2 Table — (DOCX) [file pone.0182243.s006.docx]

S9 Table. Primers for RT-qPCR analysis

| Gene | Unigene code | Accession No. | Primer sequence 5’-3’ (F/R) | Tm (°C) | Product length |
| --- | --- | --- | --- | --- | --- |
| *DXS* | CL1741 | KX056062 | GGAAGAAGAGACAAGATG/  CGAAGCAGTCATAATCAC | 80.0 | 88 |
| *ispF* | Unigene5248 | KX056066 | CCACAAGGAGACTATCAG/  CTTTGCTTTCAGGTTCAC | 80.5 | 76 |
| *ispH* | CL3923 | KX056068 | GCGGTTAGAGGATATGGAA/  AGCAGGCAGAATTACTACA | 78.0 | 97 |
| *SS* | CL11265 | KX056078 | TAGAAGCAGTTACCGAGAG/  CCTAATCCAACAAGTCCAG | 78.0 | 141 |
| *SE* | CL6504 | KU942524 | GTGGAGGAATGACTGTTG/  AGTCGTGGAGATTGTGTA | 79.0 | 78 |
| *HMGR* | CL84 | KX056076 | TGAGGATGTGGTGAAGAAG/  AGCAGAACCAGCAAGATT | 78.5 | 85 |
| *MVK* | CL10135 | KX056072 | ACACTCAGGCATCCAGAT/  GCACAATCATCCGAACCA | 79.5 | 104 |
| *DXR* | CL4453 | KX056063 | AACTGTGGACTCTGCTAC/  TATGATGGACTGCGGATG | 78.5 | 115 |
| *AACT* | CL10734 | KX056074 | TAGACAAGCAGCATTAGGT/  TGAGCAGCAAGCATAGTT | 80.0 | 102 |
| *HMGS* | CL4883 | KX056075 | AGCGAGACCGTTATAGAC/  TCCTCCATAGCAAGCATT | 79.0 | 117 |
| *ispE* | Unigene29532 | KX056065 | GCTGATGAAGTGGATATGG/  CCTCTTGCTGGTAATTCTC | 78.5 | 93 |
| *ispG* | CL4323 | KX056067 | AAGATGGACGGATGAAGT/  TCTGGAGGTTCTGTAAGG | 80.0 | 94 |
