# Supplementary material for: De novo leaf and root transcriptome analysis to identify putative genes involved in triterpenoid saponins biosynthesis in Hedera helix L
Source: PLoS One. 2017 Aug 3;12(8):e0182243. doi: 10.1371/journal.pone.0182243 (PMC5542655; doi:10.1371/journal.pone.0182243)
Supplement: S3 Table — (DOCX) [file pone.0182243.s007.docx]

S3 Table. Primer sequences for RACE clone

| Gene | Accession NO. | 5’/3’RACE primer sequence |
| --- | --- | --- |
| *HMGR* | KX056076 | ACCAGCAACAGCAGAACCAGCAAGA/  AGAGGCGGTGGAGAGGACTATGGAGAG |
| *SE* | KU942524 | TTTCTTGCCTTGCTTGGTCGGGAGATG/  TCGCTGTTTAGTTGATGTGCCTGGAC |
